# The Story of New York Briefly Told

**Published:** 1899-10

**Authors:** 


### THE STORY OF NEW YORK BRIEFLY TOLD.

THE most delightful weather greeted the arrival of members and incoming delegates as the distant portions of our country gave to New York that splendid body of veterinarians who were to actively compose the thirty-sixth annual meeting of the veterinarians of the United States, and who were to assemble for the first time as the American Veterinary Medical Association, under whose fold were to gather those who represent the progress in the all-Americas along the many lines of work and advancement that have marked the development of this the younger profession of veterinary science. Not an hour of unpleasant weather marked the period allotted for the consideration of the many important topics that brought this wonderfully large number of veterinarians together. Each day's session added largely to the number until our brethren in Gotham were exultingly joyful in that Greater New York had excelled in numbers and strength every previous meeting in the Association's history, and made future possibilities in the veterinary world, realities of progress in higher education, advancement in the sanitary field, stronger and better the work of routine practitioners, and lifted to a higher plane every aspect of the veterinarian's sphere.

In enumerating those who were there it will be noted that sectional lines were completely wiped away. The North sent their quota, the West a strong body, the South a good contingent, while the East added to this body in such goodly numbers as to make even the most regular attendants feel in a measure strangers to many faces.

*Members.* Drs. E. B. Ackerman, Brooklyn, N. Y.; John W. Adams, Philadelphia, Pa.; F. S. Allen, Philadelphia, Pa.; J. S. Anderson, Seward, Nebraska; S. Atchison, Brooklyn, N. Y.; Don C. Ayer, Omaha, Nebraska; W. L. Baker, Buffalo, N. Y.; Thos. F. Barron, Baltimore, Md.; H. W. Bath, New Brighton, N. Y.; R. R. Bell, Brooklyn, N. Y.; Geo. H. Berns, Brooklyn, N. Y.; Thomas Bland, Waterbury, Conn.; C. R. Borden, Taunton, Mass.; S. Brenton, Detroit, Mich.; John S. Buckley, Kansas City, Mo.; T. Earl Budd, Orange, N. J.; Charles Burden, New York City, N. Y.; Eugene Burget, New York City, N. Y.; C. A. Cary, Auburn, Alabama; H. A. Christmann, Milwaukee, Wis.; C. E. Clayton, New York City, N. Y.; A. W. Clement, Baltimore, Md.; W. J. Coates, New York City, N. Y.; Charles

E. Cotton, Minneapolis, Minn.; T. Bent. Cotton, Mount Vernon, Ohio; W. H. Dalrymple, Baton Rouge, La.; D. J. Dixon, Hoboken, N. J.; Wm. H. Dodge, Leominster, Mass.; Wm. Dougherty, Baltimore, Md.; G. W. Dunphy, Quincy, Mich.; Robert W. Ellis, New York City, N. Y.; J. Faust, Poughkeepsie, N. Y.; Edward C. Fox, Baltimore, Md.; H. D. Gill, New York City, N. Y.; Charles T. Goentner, Bryn Mawr, Pa.; E. A. A. Grange, Detroit, Mich.; J. O. Greeson, Kokomo, Ind.; Wm. H. Gribble, Elyria, Ohio; H. D. Hanson, New York City, N. Y.; S. J. J. Harger, Philadelphia, Pa.; R. W. Hickman, New York City, N. Y.; Charles H. Higgins, Montreal, Canada; E. H. Holden, Springfield, Mass.; W. Horace Hoskins, Philadelphia, Pa.; L. H. Howard, Boston, Mass.; R. S. Huidekoper, Washington, D. C.; John W. Jamieson, Paris, Ky.; V. L. James, Cooperstown, N. Y.; G. A. Johnson, Sioux City, Iowa; Wm. Henry Kelly, Albany, N. Y.; F. L. Killborne, Kelloggsville, N. Y.; W. B. Kille, Woodstown, N. J.; James Law, Ithaca, N. Y.; J. P. Lowe Passaic, N. J.; Wm. Herbert Lowe, Paterson, N. J.; R. P. Lyman, Hartford, Conn.; B. McInnes, Charleston, S. C.; C. C. McLean, Meadville, Pa.; L. McLean, Brooklyn, N. Y.; F. W. McLellan, Bridgeport, Conn.; James C. McNeil, Pittsburg, Pa.; F. H. Mackie, Fairhill, Md.; C. A. Mackey, New York City, N. Y.; C. J. Marshall, Philadelphia, Pa.; W. H. Martenet, Baltimore, Md.; N. S. Mayo, Storrs, Conn.; J. C. Meyer, Jr., Cincinnati, Ohio; Chester Miller, St. Louis, Mo.; W. B. E. Miller, Passaic, N. J.; John R. Mohler, Washington, D. C.; Claude D. Morris, Binghamton, N. Y.; Maurice O'Connell, Holyoke, Mass.; E. R. Ogden, Orange, N. J.; James B. Paige, Amherst, Mass.; Leonard Pearson, Philadelphia, Pa.; W. H. Pendry, Brooklyn, N. Y.; G. P. Penniman, Worcester, Mass.; Austin Peters, Jamaica Plain, Mass.; A. T. Peters, Lincoln, Neb.; B. D. Pierce, Springfield, Mass.; Lemuel Pope, Jr., Portsmouth, N. H.; M. M. Poucher, Oswego, N. Y.; H. L. Ramacciotti, Omaha, Neb.; Edward M. Ranck, Philadelphia, Pa.; James B. Rayner, West Chester, Pa.; Thomas B. Rayner, Chestnut Hill, Philadelphia, Pa.; M. H. Reynolds, St. Anthony Park, Minn.; W. L. Rhoads, Lansdowne, Pa.; Wm. H. Ridge, Trevose, Pa.; James L. Robertson, New York City, N. Y.; H. P. Rogers, Boston, Mass.; D. E. Salmon, Washington, D. C.; J. W. Schiebler, Memphis, Tenn.; Olof Schwartzkopf, Flushing, N. Y.; Walter Shaw, Dayton, Ohio; A. J. Sheldon, Boston, Mass.; E. H. Shepard, Cleveland, Ohio; Wm. C. Siegmund,

Chicago, Ill. ; D. E. Smith, Great Neck, Long Island, N. Y. ; T. E. Smith, Jersey City, N. J. ; S. Stewart, Kansas City, Kansas ; J. H. Stickney, Boston, Mass. ; W. J. Tomlinson, Williamsport, Pa. ; J. P. Turner, Washington, D. C. ; A. G. Vogt, Newark, N. J. ; Herman Wellner, New York City, N. Y. ; D. S. White, Columbus, Ohio ; W. L. Williams, Ithaca, N. Y. ; J. F. Winchester, Lawrence, Mass. ; J. M. Wright, Chicago, Ill. ; F. A. Zucker, Elizabeth, N. J.

*Members-elect.* Drs. J. M. Armstrong, Providence, R. I. ; Charles H. Cook, Rochester, N. Y. ; James W. Darby, Fort Plain, N. Y. ; John F. De Vine, Rhinebeck, N. Y. ; M. W. Drake, Philadelphia, Pa. ; Albert A. Etienne, Ware, Mass. ; W. J. Fredericks, Delawanna, N. J. ; J. T. Glennon, Newark, N. J. ; E. M. Heath, Winsted, Conn. ; Thomas J. Herr, New York City, N. Y. ; Ferd. V. Hüppe, Mt. Pocono, Pa. ; T. A. Kellar. New York City, N. Y. ; Michael Kenny, New York City, N. Y. ; A. G. Kern, Knoxville, Tenn. ; George C. Kesler, Holley, N. Y. ; J. V. Laddey, Arlington, N. J. ; Clarence Loveberry, St. Paul, Minn. ; G. W. Loveland, Torrington, Conn. ; W. J. Martin, Kankakee, Ill. ; R. C. Moore, Kansas City, Mo. ; Chester R. Perkins, Hardys, N. Y. ; E. C. Porter, New Castle, Pa. ; J. E. Ryder, New York City, N. Y. ; E. F. Sanford, Oxford, Conn. ; Wm. G. Shaw, Cincinnati, Ohio ; M. J. Tewey, Irvington-on-the-Hudson, N. Y. ; George V. Towne, Thompson, Conn. ; C. R. Witte, New Britain, Conn.

*Lady Visitors.* Connecticut—Mrs. Thomas Bland, Miss Ella Bland, Waterbury. District of Columbia—Mrs. C. B. Robinson, Mrs. J. P. Turner, Washington. Iowa—Mrs. G. A. Johnson, Sioux City. Illinois—Mrs. H. Sorby, Mrs. J. M. Wright, Chicago. Indiana—Mrs. C. B. Ainsworth, Greensburg. Kansas—Mrs. S. Stewart, Kansas City. Maryland—Mrs. A. W. Clement, Baltimore. Massachusetts—Mrs. L. H. Howard, Boston ; Mrs. W. H. Hitchings, Mrs. C. R. Simpson, Somerville ; Mrs. W. M. Simpson, Malden. Michigan—Mrs. S. Brenton, Detroit ; Mrs. J. C. Whitney, Hillsdale. Nebraska—Mrs. Don C. Ayer, Mrs. H. L. Ramacciotti, Omaha. New Hampshire—Mrs. L. Pope, Jr., Portsmouth. New Jersey—Mrs. I. N. Krowl, Newark ; Mrs. W. Herbert Lowe, Paterson ; Mrs. Grant Scott, Jersey City. New York—Mrs. E. B. Ackerman, Mrs. R. R. Bell, Mrs. George H. Berns, Miss Nellie Berns, Miss M. Ella Barlow, Mrs. F. A. Carpenter, Mrs. W. J. Cox, Mrs. Ida Donaldson, Mrs. T. Delaney, Mrs. R. W. Ellis, Mrs. M. J. Frost, Mrs. H. D. Gill, Mrs. H. D.

Hanson, Mrs. W. R. Howe, Miss Sara S. McCurdy, Mrs. R. C. Ramacciotti, Mrs. Olof Schwartzkopf, Mrs. L. Steenworth, N. Y. City; Mrs. Wm. Kelly, Miss Kelly, Albany; Mrs. D. K. Seltzer, Wellsville; Mrs. E. H. Judkins, Platz; Mrs. W. H. Salisbury, Clifton Springs; Mrs. J. F. De Vine, Rhinebeck; Mrs. Charles Cowie, Ogdensburg; Mrs. J. A. Genung, Ithaca. Ohio—Mrs. H. T. Carpenter, Ada; Mrs. T. B. Hillock, Columbus; Mrs. M. C. McLain, Jeromeville; Mrs. E. H. Shepard, Cleveland. Pennsylvania—Mrs. C. T. Goentner, Bryn Mawr; Mrs. H. Emery, Pittsburg; Mrs. A. Weir, Greenville; Mrs. C. C. McLean, Meadville; Mrs. W. Horace Hoskins, Mrs. J. T. McAnulty, Mrs. T. B. Rayner, Mrs. E. M. Ranck, Philadelphia; Mrs. R. L. Hayes, Mrs. J. B. Rayner, West Chester. Tennessee—Mrs. J. W. Schiebler, Memphis.

*Visiting Veterinarians.* Canada—Drs. W. J. Morgan, Kingston; M. H. Ten Eyck, Hamilton. Connecticut—C. L. Adams, Danielson; F. G. Atwood, New Haven; J. R. Bacon, Danbury; T. A. Donaldson, Manchester; A. A. Moody, Putnam. District of Columbia—C. B. Robinson, Washington. Georgia—C. R. Jolly, Atlanta. Illinois—C. P. Lovejoy, State Veterinarian, Princeton. Indiana—C. B. Ainsworth, Greensburg; J. Beatty, Indianapolis. Iowa—W. R. Fullerton, Dubuque; R. J. Scott, Independence. Maine—F. E. Freeman, Rockland; F. W. Huntington, G. F. Westcott, Portland; A. Joly, Waterville; J. D. Salley, Skowhegan; W. L. West, Belfast. Massachusetts—F. Abele, Jr. Quincy; W. H. Hitchings, Boston; A. G. Potter, Adams; C. R. Simpson, Somerville; W. N. Simpson, Malden; C. H. Tilton, Ashland; J. A. Vilas, Lowell. Michigan—J. C. Whitney, Hillsdale. Nebraska—V. C. Barber, Lincoln. New Jersey—T. J. Cooper, Paterson; Wm. P. Fink, Atlantic City; Wm. Gall, Matawan; G. F. Harker, Trenton; R. O. Hasbrouck, Passaic; J. D. Hopkins, Newark; L. D. Horner, Woodstown; I. N. Krowl, Monticello; F. L. Stevens, Union Hill. New York—H. Amling, Jr., C. S. Atchison, T. Delaney, Robert Dickson, T. H. Doyle, A. Eickhorn, J. H. Ferster, H. C. Glover, W. R. Howe, R. S. MacKellar, H. K. Miller, W. C. Miller, J. J. Murray, B. Pendry, E. L. Sanders, T. G. Sherwood, W. J. Taylor, J. Weiss, S. West, G. W. Meyer, New York City; M. T. Brewster, Emil Knight, A. G. Tegg, Rochester; T. J. Cooper, H. D. Mayne, Malone; Charles Cowie, Ogdensburg; E. M. Casey, Oxford; G. C. Dewitt, Oak Hill; E. H. Judkins, Platz; F. D. Markham, Port Leyden; T. Meredith, Jamestown; R. Perkins, Hardys;

W. H. Salisbury, Clifton; D. K. Seltzer, Wellsville; J. W. Taylor, Henrietta; H. A. Turner, Syracuse; F. O. Wright, White Plains; J. H. Youngs, Belvidere. Ohio—H. T. Carpenter, Ada; P. A. Dillahunt, T. B. Hillock, Columbus; J. D. Fair, Berlin; W. C. Fair, Cleveland; R. G. Holland, Wellington; M. C. McLain, Jeromeville. Pennsylvania—E. M. Conard, West Grove; J. T. McAnulty, W. L. Zuill, Philadephia; C. W. Boyd, J. S. Lacock, Allegheny; H. Emery, Wilkinsburg; J. Helmer, Scranton; L. E. Mead, Tunkhannock; W. A. Meredith, Corry; B. F. Minick, Columbia; J. H. St. Clair, Indiana; H. W. Turner, Lahaska; B. M. Underhill, Media; A. W. Weir, Greenville; J. C. Foelker, Allentown; W. J. Fretz, Perkasie. Vermont—C. W. Fisher, Burlington. U. S. A.—R. B. Corcoran.

*Delegates.* Pennsylvania State Veterinary Medical Association, J. C. Foelker, Allentown; Ontario Veterinary College, D. King Smith, M.D., Toronto, Canada.

*Other Visitors.* J. T. Connors, M.D., Boston, Mass.; Prof. S. H. Gage, Prof. V. A. Moore, Ithaca, N. Y.; S. K. Spaulding, M.D., Omaha, Neb.; J. P. Lott, Springfield, Ill.; H. Sorby, Chicago, Ill.; C. E. Dornheim, Providence, R. I.; G. W. Smith, R. R. Agt.; M. Hallanan, New York City, N. Y.; R. L. Hayes, West Chester, Pa.

A railroad accident in Northern New York made the arrival of Secretary Stewart on Monday, September 4th, several hours late, and the meeting of the Executive Committee was necessarily postponed until evening. The growth of work for this body and the wisdom of the founders of the Association in assigning to it the general responsibility for the management of Association affairs, were never better exemplified than in New York, for had the volume of work allotted to this body fallen to the lot of the Association its sessions would have been entirely taken up in its disposal and the consideration of papers and reports relegated to another year. Each day this body met in advance of the general sessions, and its many recommendations here recorded will afford some estimate of the great amount of work transacted. Happily for the members of this executive body the Secretary had printed on slip form much of the business it was to consider, and its disposition was much facilitated thereby.

The opening session on the morning of the 5th found that veteran member of the profession and of this organization, Prof. James L. Robertson, peculiarly happy in his address of welcome on behalf of Greater New York. To this warm welcome Dr. D. E. Salmon fittingly replied, and touched upon the rapid growth of the organization during the last ten years.

*President's Address.* President Clement followed in his annual address, referring very warmly to the wisdom that prevailed among those who founded this organization in the establishment of wise rules for the guidance and conduct of its members. He briefly referred to the rapid studies of advancement in veterinary surgery and the triumphs of certain operations for conditions that formerly consigned these animals to days of uselessness or the knacker's yard. Comparing the work of the veterinarian with the practitioner of human medicine, he considered it more interesting and on the average quite as profitable.

The much-vaunted horseless age did not meet with much encouragement on his part, as he noted the field that the horse had occupied in all ages and among all peoples. He spoke hopefully of still further attainments in army legislation and the ultimate securing of a veterinary corps, to which he urged continued effort and work. Speaking of the Department of Agriculture and the Bureau of Animal Industry, he fittingly referred to its chief, Dr. D. E. Salmon, as one who had done good work, not only for the profession, but for the army and the country at large.

*Executive Committee's Recommendations.* The several recommendations of the Executive Committee were then acted upon in order.

The Association accepted the requested resignations of Drs. Kinnell, of Massachusetts; Ogle and McLean, of New York; Collasowitz, of Missouri, and H. D. Fenimore, of Knoxville, Tennessee. A desire was expressed to retain the membership of Dr. Dinwiddie, and a committee was appointed to confer with him.

The charges (violation of code of ethics) against Dr. C. J. Sihler were dismissed on evidence of their having been discontinued. Those against Dr. Emele Pouppirt, in advertising contrary to the code of ethics, were sustained, and his expulsion followed.

Favorable action did not prevail in the interest of certain graduates of two-year colleges, during 1897 and 1898, that had since extended their curriculum to the standard of requirements of the Association, though it had the favorable recommendation of the Executive Committee.

The proposed defining of the status of agricultural college graduates and students in veterinary colleges, after an earnest and wide range of discussion, was referred back to the Executive Committee.

The restoration to membership of Drs. R. S. Huidekoper and H. P. Rogers was agreed to.

*Elected to Membership.* The following applicants, whose names were favorably recommended by the Executive Committee, were elected to membership:

J. M. Armstrong, M.D.V. (Harvard, 1896), Providence, R. I.; voucher, R. P. Lyman.

Albert Babb, M.D.C. (C. V. C., 1893), Springfield, Ill.; voucher, E. M. Nighbert. S. L. Blount, V.M.D. (V. D. U. of Pa, 1898), Philadelphia, Pa.; vouchers, Leonard Pearson and W. Horace Hoskins. F. E. Burnham, D.V.S. (C. V. C., 1890), West Superior, Wis.; voucher, R. H. Harrison. George W. Butler, V.S. (Ont. V. C., 1884), Milwaukee, Wis.; voucher, R. H. Harrison.

Thomas S. Childs, V.S. (N. Y. C. V. S., 1891), Saratoga Springs, N. Y.; voucher, Wm. H. Kelly. Charles H. Cook, V.S. (Ont. V. C., 1872), Rochester, N. Y.; voucher, Wm. Henry Kelly.

James W. Darby, V.S. (Ont. V. C., 1890), Fort Plain, N. Y.; voucher, Wm. Henry Kelly. John F. De Vine, D.V.S. (A. V. C., 1898), Rhinebeck, N. Y.; vouchers, Wm. Henry Kelly and W. J. Coates. M. W. Drake, D.V.S. (A. V. C., 1890), Philadelphia, Pa.; vouchers, W. H. Ridge and J. Payne Lowe. Henry W. Dustan, D.V.M. (N. Y. S. V. C., 1898), Morristown, N. J.; voucher, W. L. Williams.

Albert A. Etienne, D.V.S. (Laval Univ., 1890), Ware, Mass.; vouchers, J. F. Winchester and B. D. Pierce.

Pierre A. Fish, D.V.M. (N. Y. S. V. C., 1899), Ithaca, N. Y.; vouchers, James Law and W. L. Williams. Wm. J. Fredericks, V.S. (N. Y. C. V. S., 1895), Delawanna, N. J.; voucher, J. Payne Lowe.

James T. Glennon, V.S. (N. Y. C. V. S., 1896), Newark, N. J.; vouchers, A. G. Vogt and J. Payne Lowe. J. N. Gould, M.D.C. (C. V. C., 1893), Worthington, Minn.; vouchers, M. H. Reynolds and S. Stewart.

C. A. Hamblet, V.M.D. (V. D. U. of Pa., 1897), Lowell, Mass.; voucher, J. F. Winchester. E. M. Heath, D.V.S. (C. V. C., 1887), Winsted, Conn.; voucher, Richard P. Lyman. W. A. Heck, D.V.M. (I. A. C., 1891), Sioux City, Ia.; vouchers, S. Stewart and G. A. Johnson. Samuel G. Hendron, V.M.D. (V. D. Univ. of Pa.), Indianapolis, Ind.; voucher, Tait Butler. T. J. Herr, D.V.S. (A. V. C., 1879), New York

City, N. Y.; vouchers, James Law and C. E. Clayton. Herbert Hoopes, V.M.D. (V. D. U. of Pa., 1899), Bynum, Md.; vouchers, A. W. Clement and Leonard Pearson. Benjamin F. Hoover, V.S. (Ont. V. C., 1895), Davis, Ill.; voucher, E. M. Nighbert. Ferd. V. Hüppe, V.S. (N. Y. C. V. S., 1892), Mt. Pocono, Pa.; voucher, Wm. Henry Kelly.

M. Jacobs, V.M.D. (V. D. U. of Pa., 1899), Philadelphia, Pa.; vouchers, Leonard Pearson and C. J. Marshall.

Theo. A. Keller, D.V.S. (A. V. C., 1892), New York City, N.Y.; vouchers, E. B. Ackerman and J. L. Robertson. A. B. Kelly, D.V.M. (N. Y. S. V. C., 1898), Albany, N. Y.; voucher, Wm. Henry Kelly. Michael Kenny, V.S. (N. Y. C. V. S., 1888), New York City, N. Y.; vouchers, H. D. Gill and James Law. George C. Kesler, V.S. (Ont. V. C., 1892), Holley, N. Y.; voucher, Wm. Henry Kelly. A. J. Kline, V.S. (Ont. V. C., 1894), Wauseon, Ohio; voucher, T. Bent. Cotton.

John T. Lee, D.V.S. (A. V. C., 1889), Tacoma, Washington; voucher, S. B. Nelson. A. G. Kern, V.M.D. (V. D. U. of Pa., 1899), Knoxville, Tenn.; vouchers, Leonard Pearson and W. Horace Hoskins. Henry S. Lewis, M.D.V. (Harvard, 1882), Chelsea, Mass.; vouchers, Wm. Stinson and B. D. Pierce. J. V. Ladley, D.V.S. (A. V. C., 1897), Arlington, N. J.; voucher, J. Payne Lowe. G. W. Loveland, M.D.C. (C. V. C., 1894), Torrington, Conn.; voucher, Richard P. Lyman. Clarence Loveberry, V.S. (V. D. Ohio S. U., 1895), St. Paul, Minn.; voucher, W. Horace Hoskins.

Wm. McLean, V.S. (Ont. V. C., 1882), Portland, Oregon; voucher, S. B. Nelson. John H. McNeall, V.M.D. (V. D. U. of Pa., 1898), Buffalo, N. Y.; vouchers, Leonard Pearson and C. J. Marshall. W. J. Martin, M.D.C. (Chicago V. C., 1897), Kankakee, Ill.; vouchers, R. R. Bell and W. Horace Hoskins. J. N. Megary, V.M.D. (V. D. U. of Pa., 1898), Baltimore, Md.; vouchers, A. W. Clement and W. H. Martenet. W. T. Monsarrat, V.S. (Ont. V. C., 1889), Honolulu, H. I.; vouchers, S. Stewart and R. R. Bell. R. C. Moore, D.V.S. (C. V. C., 1887), Kansas City, Mo.; voucher, S. Stewart. John J. Moynahan, D.V.S. (A. V. C., 1895), Holyoke, Mass.; vouchers, B. D. Pierce and H. E. Holden. M. J. Myers, V.S. (O. S. U., 1897), St. Louis, Mo.; vouchers, C. Miller and Charles Ellis.

James D. Nighbert, V.S. (Ont. V. C., 1889), Pittsfield, Ill.; voucher, E. N. Nighbert.

C. H. Parkinson, D.V.S. (Columbia V. C., 1881), Portland,

Conn.; voucher, Richard P. Lyman. Charles R. Perkins, D.V.M. (N. Y. S. V. C., 1899), Hardys, N. Y.; vouchers, James Law and W. L. Williams. E. C. Porter, V.S. (Ont. V. C., 1891), New Castle, Pa.; vouchers, C. C. McLean and W. Horace Hoskins.

A. G. G. Richardson, V.M.D. (V. D. U. of Pa., 1894), Cambridge, Mass.; vouchers, A. J. Sheldon and J. P. O'Leary. J. E. Ryder, D.V.S. (A. V. C., 1884), New York City, N. Y.; vouchers, W. H. Pendry and H. D. Hanson.

E. F. Sanford, D.V.S. (A. V. C., 1898), Waterbury, Conn.; vouchers, Thomas Bland and C. E. Clayton. Wm. G. Shaw, V.M.D. (V. D. U. of Pa., 1897), Cincinnati, Ohio; vouchers, Leonard Pearson and W. Horace Hoskins. R. V. Smith, V.S. (Ont. V. C., 1888), Baltimore, Md.; vouchers, A. W. Clement and F. H. Mackie.

Martin J. Tewey, D.V.S. (A.V.C., 1889), Irvington-on-the-Hudson, N. Y.; voucher, Wm. Henry Kelly. George V. Towne, D.V.S. (A. V. C., 1890), Thompson, Conn.; voucher, Richard P. Lyman. R. W. Tuck, V.S. (Ont. V. C., 1892), Indianapolis, Ind.; voucher, Tait Butler.

S. F. Wadsworth, M.D.V. (Harvard Vet. Dept., 1899), Keene, N. H.; voucher, Richard P. Lyman. C. R. Witte, D.V.S. (A. V. C., 1896), New Britain, Conn.; vouchers, James Law and H. D. Hanson.

The application of Dr. James H. Young, graduate of the Ohio Veterinary College, was rejected.

The applications of Drs. J. J. Riordan and T. S. Childs were held over pending abatement of forms of advertising conflicting with the code of ethics. Favorable action followed as to the application of Dr. Childs, on assurances that he would fully comply with all the requirements.

*Publication Committee.* Chairman Williams, of this committee, briefly reported the work done during the past year, and, also as Librarian, of the number of copies of the Association proceedings in his keeping, and received a hearty vote of thanks for the excellent work done.

*The Committees on Intelligence and Education and Diseases* were not prepared to report, and for the first time in many years these fields of work were not covered by those to whom the tasks were allotted.

*Army Legislation.*[1] Chairman Salmon, of this committee, fully

[1] See page 598.

reported the work of this body during the past year, and most feelingly referred to the great loss sustained by the death of Army Veterinarian Dr. M. J. Treacy.

*Local Committee of Arrangements.* Drs. Gill and Bell, of the Local Committee of Arrangements, were so much overcome by the large and generous acceptance of their invitations to everybody to come to Gotham and have the best time in their lives, that visions of a shortage of supplies at the clam-bake and of an overloaded boat caused beads of sweat to mar their usually calm and placid visages; but "all's well that ends well," and every feature of pleasures planned was more than carried out, and everyone had a good word for the local Committee of Arrangements.

*Treasurer's Report.* This report proved a gratifying one, and Treasurer Lowe, who held the purse-strings for the past year, had faithfully husbanded the Association's resources, and in his usual business-like manner treated of the receipts and disbursements in a complete manner. Some $1553.73 were placed with him during the year, and bills were liquidated to the extent of $863.94, leaving a balance in the treasury of $689.79. The report was received and referred to the Auditing Committee.

*Secretary's Report.* Secretary Stewart, whose years of continued efficient service were commenced anew at the close of the New York meeting for he has made a record of being everlastingly at it that has contributed very much indeed to the success and progress of the organization—presented a very encouraging report, showing collections from dues, etc., of $1248.40, and expenditures of $1182.93. The standing of the members, in view of the fact of a reduction of 40 per cent. in dues charged, was not as favorable as was to be expected, as fifty-three members still owe more than two years' dues, fifty-three others owe just two years, and fifty-five members owe less than two years, or some one hundred and sixty-one members, more than one-third of the total membership, whose accounts are open and which should have been liquidated. The Association has need of every dollar of its prospective income for the various aspects of its work, and Secretary Stewart has been untiring in urging the members to prompt payment.

*State Secretaries' Reports.* A number of communications from State Secretaries were received from Arizona, New Mexico, and California; the latter indulging in the hope, long deferred, that the Association might soon plan for a meeting on the Pacific coast.

From Connecticut, reviewing efforts at legislation there, pointing to the lack of aggressive association work in that State as one of the causes of failure.

From Delaware, covering the field of contagious and infectious diseases, and paying a splendid compliment to the evidences on all sides of broader knowledge in the care and prevention of many of the ailments and misfortunes of animals due in the past to ignorance.

For the District of Columbia and their needs of better legislation for proper food inspection, a speedway for owners of pleasure horses, and many other regulations of a sanitary nature.

From New Hampshire, which will in the future join with Vermont in veterinary organization. A reference to the dangerous conclusions arrived at by a body of laymen in conducting a limited investigation in dealing with tuberculosis among cattle.

Of South Carolina, the absence of any State laws of value in dealing with animal diseases with reference to the prevalence of the so-called "dumb rabies."

Tennessee reported the lack of support given legislation desired for the regulation of the practice of veterinary science. The prevalence of Texas fever referred to; the erection of a large public abattoir in Nashville; the small percentage of tuberculosis found among animals tested was favorably commented upon; the greater interest in the State veterinary organization since the meeting of the national organization at Nashville.

From Wisconsin was reported a general improvement all along the lines of veterinary work; a movement in Milwaukee looking to a tuberculin test of all milk cattle whose products are sold in that city; an outbreak of anthrax at a tannery, costing six men's lives, from imported diseased hides.

The afternoon session was occupied by an excellent paper by Dr. R. R. Bell on "Acetanilid as an Antipyretic."

The subject of "Municipal Meat-inspection" was introduced by a paper read by Dr. E. B. Ackerman.

Drs. J. P. Turner, L. MacLean, Stewart, Lowe, and Kenny discussed the value and mode of use of acetanilid, while the subject of municipal meat-inspection was considered by Drs. John Faust, Salmon, Dunphy, Austin, Peters, Gill, G. A. Johnson, and Lowe.

(Continued in the November number.)

---

Drs. William Sheppard, Thomas G. Sherwood, and J. E. Ryder will officiate as veterinary inspectors at the national horseshow in Madison Square Garden, New York, in November.

